# Perceived Support from Best Friends and Depressive Symptoms During Adolescence: Disentangling Personal from Dyadic Level Effects

**DOI:** 10.1007/s10802-022-00999-x

**Published:** 2022-12-19

**Authors:** Steffie van der Mey-Baijens, J. Marieke Buil, Patricia Vuijk, Kim C. M. Bul, Susan Branje, Wim Meeus, Pol A. C. van Lier

**Affiliations:** 1 Research Centre Urban Talent, Rotterdam University of Applied Sciences, Rochussenstraat 198, 3015 EK Rotterdam, the Netherlands; 2 Department of Clinical, Neuro and Developmental Psychology, Vrije Universiteit Amsterdam, Amsterdam, the Netherlands; 3 Institute for Health and Wellbeing, Centre for Intelligent Healthcare, Coventry University, Coventry, UK; 4 Department of Youth and Family, Utrecht University, Utrecht, the Netherlands; 5 Amsterdam Public Health Research Institute – Mental Health, Amsterdam, the Netherlands

**Keywords:** Social support, Friendship, Multilevel analyses, Adolescence, Depressive symptoms

## Abstract

Support from best friends is an important interpersonal factor in adolescent depression development but is often studied from an individual perspective in which dyadic effects are overlooked. This study aims to a) test whether differences in support vary at the individual level and are related to individual differences in the development of depressive symptoms, whether these differences vary at the dyadic level and are related to dyadic depression symptom development, or both, b) explore whether these associations are moderated by initial levels of depressive symptoms on the individual and/or dyadic level. Data from 452 adolescents (*M*age = 13.03), nested in 226 same-gender friendship dyads (60.6% boy-dyads) who participated in the RADAR-Y project were included. Best friends self-reported annually (2006–2008; 3 waves) on their own depressive symptoms and perceived support from their friend. Multilevel models showed no direct association between support and depression development on the individual or dyadic level. However, the initial level of dyads’ depressive symptoms moderated the association between dyadic support and dyads’ subsequent depression symptom development. When dyads experienced relatively more initial depressive symptoms, higher levels of dyadic support were associated with relative increasing dyadic depressive symptoms. When dyads experienced relatively few initial depressive symptoms, higher levels of dyadic support were associated with relative decreasing dyadic depressive symptoms. Findings suggest that support from best friends can either protect against or exacerbate the development of depressive symptoms for friends, depending on the initial level of depressive symptoms of the dyad.

**Supplementary Information:**

The online version contains supplementary material available at 10.1007/s10802-022-00999-x.

The prevalence of depressive symptoms peaks in early to middle adolescence; almost 12.5% of young adolescents experience depressive symptoms and over 4% have clinically elevated levels of depressive symptoms in this period (Costello et al., 2006; Keyes et al., 2019). Depressive symptoms, such as dysphoric mood and negative self-evaluation, are associated with significant impairments in a range of domains, such as academic performance, social functioning, physical health, and well-being (Clayborne et al., 2019; Jaycox et al., 2009). Support from friends is an important interpersonal factor associated with depressive symptoms development during adolescence (Hankin et al., 2018; Hartup, 1993; Helsen et al., 2000; Young et al., 2005). Previous studies have mainly investigated the association of social support with depressive symptoms from an individual perspective. However, as support within friendships by nature is an interpersonal quality that is the result of reciprocal social behavior and dyadic interactions between two adolescents, friend support may be (at least in part) a dyadic process experienced by the dyad (Turner & Brown, 2010). The same holds true for depressive symptoms within dyadic friendships (Boersma-van Dam et al., 2019; Brechwald & Prinstein, 2011; Giletta et al., 2011), where dyad-members as a pair might differ in their development of depressive symptoms from other dyads. Nonetheless, disentangling individual from dyadic effects is often overlooked in previous studies focusing on the association between friend support and depressive symptoms. We used a longitudinal multilevel design following 452 adolescents nested in 226 same-gender friendship dyads across two years (3 waves) to test whether the association of support by best friends and subsequent depressive symptom development should be regarded as an individual process, a dyadic process, or both.

## Friend Support and Depressive Symptoms

During the transition from childhood to adolescence, young people become increasingly autonomous from their parents while they at the same time build close emotional bonds with their peers (Brown & Larson, 2009; Howes & Aikins, 2002). During this developmental period, adolescent friendships often become a highly relevant, if not the primary, source of social support (Bokhorst et al., 2010; Furman & Buhrmester, 1992). High levels of perceived support from peers are generally associated with positive psychological states, such as higher self-worth and positive affect, as well as reduced negative affect (Chu et al., 2010; Cohen & Wills, 1985; Malecki & Demary, 2002). Indeed, a recent U.S. longitudinal survey study (Finan et al., 2018) and comprehensive meta-analysis (Rueger et al., 2016) found that support from close friends aged 12 – 19 years was associated with lower levels of depressive symptoms concurrently as well as over time, although weakly. In contrast, several recent studies varying from cross-sectional to longitudinal survey studies and covering the complete age range of adolescence (resp. 14–19 years; 15–17 years and 12–15 years), across cultures failed to find associations between close friend support and depressive symptoms (Bámaca-Colbert et al., 2017; Ling et al., 2022; Lyell et al., 2020). To better understand the conditions under which friend support is associated with depressive symptoms, we suggest that two considerations should be taken into account: the dyadic nature of best friend support and initial depression levels.

## The Dyadic Nature of Best Friend Support

The first consideration entails the dyadic nature of the association between perceived support from best friends and depressive symptoms development. Most prior studies investigating the association between best friend support and the development of depressive symptoms in adolescence focused on individuals as the unit of study. Therefore, these studies did not test whether the association between perceived support and depressive symptoms mostly influences individual adolescents, the friend-dyad as a couple, or both. However, best friend support by definition includes two individuals (i.e., the two friends), who in addition to their personally experienced support by their friend, likely share these experiences to some extent, referred to as a ‘dyadic effect’. The same holds for depressive symptom development.

Several mechanisms might explain why perceived support and experienced depressive symptoms may become shared within friendships. For instance, friends might clique together because they both appreciate support by friends in their handling of their mental state of depression, and regard support as an important friendship quality. Similarly, friends might pick each other out because they both experience depressive symptoms and appreciate mutual understanding (i.e., peer selection effects). Indeed, a previous study found support for this type of selection effects (Van Zalk et al., 2010). In addition, and not necessarily mutually exclusive from the processes of selection, it has been found that friends with depressive symptoms become more alike over time in their depression levels (Boersma-van Dam et al., 2019; Brechwald & Prinstein, 2011; Brown & Larson, 2009; Giletta et al., 2011); a process known as peer evocation or ‘spill over’ effects. Thus, within best friend dyads, the shared experience of friend support may affect the course of their depressive symptom development for both friends together. In addition, there is emerging evidence that some friendship dyads use specific communication patterns such as co-rumination—the tendency to dwell on problems and focus on negative emotions when discussing problems – that may cause negative affect to get worse instead of alleviated (Rose et al., 2007, 2014).

## The Role of Initial Levels of Depressive Symptoms

The second consideration is that the association between support and subsequent depressive symptoms might depend upon the initial levels of depressive symptoms of the adolescent, or the friendship dyad (Gariepy et al., 2016; Stice et al., 2004). Specifically, previous studies have suggested that adolescents with only a few depressive symptoms may benefit from seeking support from friends to alleviate their symptoms, while adolescents who show more or clinical levels of depressive symptoms might not benefit from peer support and may be in need of professional healthcare treatment (Gariepy et al., 2016; Pfeiffer et al., 2012). That is, mutual support seeking might decrease depressive symptoms when these symptoms are mild. However, when support seeking becomes excessive and when friends start to engage in behaviors like co-rumination, support seeking has been associated with increases in adolescent depression development (Rose, 2002; Rose et al., 2007). An observational study on co-ruminative conversations between adolescent friend-dyads found that particularly the dwelling on problems and excessive focus on negative emotions when discussing problems was linked to internalizing problems (Rose et al., 2014).

Empirical tests of whether the association between friend support and depressive symptoms differs for adolescents who experience more versus few initial depressive symptoms are as of yet scarce. One previous study that focused on potential differential effects of support for high-risk (defined as adolescents who were diagnosed with a psychiatric condition) versus low-risk adolescents (adolescents without a psychiatric diagnosis) found that the strength of the association between support and depressive symptoms did not differ between these two groups (Rueger et al., 2016). Nevertheless, a recent meta-analysis emphasized the need for more empirical research into the ‘initial symptom level’ hypothesis (Pfeiffer et al., 2012).

## Disentangling Individual from Dyadic Level Effects in the Support – Depression Link

When studying the role of support in predicting depressive symptoms within friendship dyads, and the possible moderation effect of initial levels of depressive symptoms in this association, four options should be considered. The first option (hypothesis 1) proposes a simple main effects model where best friend support associates with the subsequent development of depressive symptoms, and that these associations might act on the individual level, on a dyadic level, or on both levels. In this model (a two-level model with a within, i.e., individual and a between, i.e., dyadic, component; Kenny et al., 2020; Ledermann & Kenny, 2017), a pure individual effect indicates that the association between support and depression development appears on the individual level only and that there are no additional dyadic effects. The opposite holds true when a pure dyadic effect is found. When both an individual effect as well as a dyadic effect is found, this indicates that the association between support and depression development is best understood as a partial individual and a partial dyadic process.

Alternative to this main effect option, three moderation options are possible (hypotheses 2A-2C). Hypothesis 2A proposes that the association between individually perceived support and individual depressive symptoms depends on the initial levels of depressive problems of the individual adolescent (moderation at the within-level). Here, again, the association between best friend support and depression development is best understood as an individual process, but this model adds that the initial level of depression matters too. Hypothesis 2B proposes that the association between perceived support and experienced depressive symptoms of both adolescents in the dyadic friendship might depend on the initial level of depressive symptoms experienced by the dyad as a whole. Hypothesis 2C, lastly, proposes that the association between individually perceived support and subsequent individual depressive symptoms depends on the levels of depressive symptoms experienced by the dyad as a whole. This would suggest a cross-level moderation where shared initial levels of depressive symptoms of the dyads influence the strength of the association between support and subsequent depressive symptoms for the two individual adolescents within the dyad differently. When such a cross-level interaction is found, follow-up studies should focus on which individual characteristics might explain why some adolescents are more susceptible to the influence of perceived friend support than their friend regarding their depression development, while both friends experience similar initial levels of depressive symptoms.

## Present Study

Using a longitudinal, multi-level study in which adolescents were followed over two years, the aim of the present study was to test if support from best friends is associated with the development of depressive symptoms over time, either at the individual level, dyadic level, or both (hypothesis 1). Furthermore, we examined whether these potential associations depended upon initial levels of depressive symptoms and at which level (hypotheses 2A-2C), as has been suggested in previous studies (Gariepy et al., 2016; Pfeiffer et al., 2012).

Gender was included as a covariate in all tested models as there is mixed empirical evidence on the differential levels of and associations between friend support and depressive symptoms for boys and girls. For example, some studies reported that girls experience more support from close friends than boys (Rose & Rudolph, 2006; Rueger et al., 2010), other studies found no gender differences regarding friendship satisfaction and stability (Rose & Asher, 2017). Some studies found that higher levels of support were associated with fewer depressive symptoms, but particularly for girls (Chu et al., 2010; Rueger et al., 2010), while others find little to no gender differences in this association (Gregory et al., 2020; Rueger et al., 2016). Lastly, communication strategies that might underly a positive association between friend support and subsequent depressive symptom development, such as co-rumination, might be particularly characteristic for girls (Hankin et al., 2010; Rose, 2002; Rose & Rudolph, 2006; Rose et al., 2007, 2014).

Based on previous empirical work, we expected that higher levels of perceived support would be associated with fewer depressive symptoms over time for individual adolescents (i.e., a within-level effect; Rueger et al., 2016). Furthermore, based on the emerging evidence that both friendship support and subsequent depressive symptoms may yield – to some degree – dyadic effects, we also expected to find associations at the dyadic level (Boersma-van Dam et al., 2019; Brechwald & Prinstein, 2011; Brown & Larson, 2009; Giletta et al., 2011). Lastly, given the – to our knowledge – lack of previous empirical work on initial depression levels as a potential moderator in the association between support and subsequent depressive symptoms, we could not formulate an a-priori hypothesis regarding a potential moderation effect and the direction of such an effect.

## Method

### Participants

We selected our sample of 226 target adolescents and their best friends (*N* = 452; *N* = 226 dyads) from a total of 497 target adolescents and their best friends who participated in the Dutch Research on Adolescent Development And Relationships-Young (RADAR-Y) research project (Branje & Meeus, 2018). In this longitudinal cohort study, data of Dutch adolescents, their family members, and their friends were collected annually (one-year intervals) over six years. Data of the RADAR project is stored in DANS, ‘*the Dutch national centre of expertise and repository for research* *data’*, and available via Research on adolescent development and relationships (young cohort) - EASY (knaw.nl).

In the current study, we used data of the early adolescence period covering three measurement waves that were collected from 2006 till 2008. Adolescents were included in the present study when they participated with the same best friend in all three measurement waves (*n* = 244). Adolescents could invite a friend from another gender, who was not a romantic partner, as their best friend (*n* = 8 pairs). However, due to the small number of mixed-gender friendships and potential differential effects for mixed- versus same-gender friendships (Kuttler et al., 1999), mixed-gender friendships were excluded in the present study. Furthermore, participants with missing data on support or depressive symptoms were excluded (*n* = 9), resulting in a total sample of 452 adolescents and friends (274 boys; 137 boy-pairs; 60.6%). At the first measurement wave, adolescents were on average 13.03 years old (*SD* = 0.47 years). Over 95% of the included adolescents had a native Dutch background, five adolescents reported having a western background other than Dutch (1%), and 14 adolescents stated to have a non-western background (3%). Approximately 93% came from families with a medium to high socioeconomic status (SES). This is higher than the socioeconomic position of the average Dutch population where 70.5% has a medium to high SES (Statistics Netherlands, 2022).

Results from independent samples *t*-tests indicated that at the first measurement wave adolescents included in our sample had slightly lower levels of depressive symptoms (*M* = 14.20, *SD* = 10.47) compared to adolescents who were excluded from our sample (*M* = 15.93, *SD* = 11.39; *t*(932.716) = -2.42, *p* < 0.05, Cohen’s *d* = 0.15). No differences in levels of perceived friend support between the included and excluded group were found at the first wave (*p* = 0.92).

### Procedure

Adolescents were recruited from the middle and western parts of the Netherlands. Both parents and adolescents provided active written informed consent at each measurement wave and were informed that they could withdraw their consent for participation at any time during the study. At each wave the target adolescent, both parents and at least one sibling were invited to participate. In addition, participants were asked to invite their best friend to participate in the study as well. The data were collected by trained research assistants who visited the adolescents every year at home. After receiving verbal and written instructions, adolescents were asked to fill out questionnaires on paper. The family received a gratitude for participating worth of €100 each home visit and adolescents’ best friends received €25 as an additional reward for their participation. The procedures and measures used were all approved by the Medical Research Ethics Committee of the University Medical Center Utrecht (RADAR: Research on Adolescent Development and Relationships, 05/159-K). A detailed description of the complete study design and sample of the RADAR project can be found elsewhere (Van Lier et al., 2008).

### Measures

#### Best Friend Support

The ‘support’ subscale of the Network of Relationships Inventory (NRI) assessed best friend support (Furman & Buhrmester, 1992). The subscale consists of eight items. Adolescents and their best friend were instructed to take each other in mind while answering items such as: ‘How much does your best friend really care about you?’, ‘How often do you play around and have fun with this person?’ and ‘How much does this person help you figure out or fix problems?’. Responses were rated on a 5-point Likert-scale 0 (little or none) to 4 (the most) and were summed into a total support score. Higher scores refer to higher levels of support. The NRI shows good psychometric properties in adolescent samples, such as high internal consistency (α = 0.68 to α = 0.95) and factor loadings ranging from 0.30 to 0.90 (Ackermann et al., 2018; Furman & Buhrmester, 2009). Cronbach’s alphas in the current study ranged from 0.80 to 0.89 across assessments.

#### Depressive Symptoms

The Reynolds Adolescent Depression Scale-2nd edition (Reynolds, 2004) was used to assess adolescent depressive symptoms. The scale included in our study consists of 23 items, and has the following three subscales: dysphoric mood, negative self-evaluation, and somatic complaints. The subscale anhedonia is excluded in our study. Example items of the three subscales include ‘I feel sad’, ‘I feel I am no good’ and ‘I have trouble sleeping’, respectively. However, to calculate the percentage of adolescents who experience clinical levels of depression in wave 1, the full depression scale including all 4 subscales was used. Adolescents were instructed to respond to items in a way that best described how they feel on a 4-point scale, ranging from 0 (almost never) to 3 (most of the time). Items from each scale were summed into a total depression score. Previous studies have reported good psychometric properties, such as high internal consistency (α = 0.94; 95% CI = 0.93–0.95; average interitem r = 0.34) and scale reliability of 0.85 (95% CI = 0.82–0.89) (Myers & Winters, 2002; Osman et al., 2010). Across the three measurement waves, Cronbach’s alphas in the current study ranged from 0.91 to 0.94 across assessments. Furthermore, in the first measurement wave 7 adolescents (2.7%) had a raw score of 76 or above on the depression scale (RADS-2) indicating that they experience clinical levels of depression. For all three waves boys experienced fewer depressive symptoms and lower levels of support than girls (see Online Resource 1).

### Data Analyses

To examine the nature of the association of friendship support nested in friendship-dyads on subsequent depressive symptoms, we used multilevel models using Mplus version 8.5 (Mplus code is available in the Open Science Framework; OSF, via the following link: bit.ly/3UFPEVj) (Kenny & Kashy, 2014; Muthén & Muthén, 2017). In this multilevel model, associations between perceived support and depressive symptoms can be decomposed in variance on the individual (within) and dyadic/friendship (between) level (Kenny & Kashy, 2014). The intraclass correlations (ICCs) was estimated for depression and support for all three waves to describe the intra-dyad homogeneity (i.e., the between-dyad variability relative to the total variation) on both variables (Doğan & Doğan, 2015). The higher the ICC, the more between-dyad variation (i.e., scores of dyads differ from each other) and the more intra-dyad homogeneity (i.e., adolescents within the dyads score similarly). To this end, an empty model (model 0) was specified in which only the variances and the means of support and depressive symptoms were estimated, but no structural model was specified. As dyads consist of two best friends with no explicit characteristics that differentiate them (e.g., gender), they are regarded indistinguishable dyads in the models (Kenny & Kashy, 2014).

**Fig. 1:**
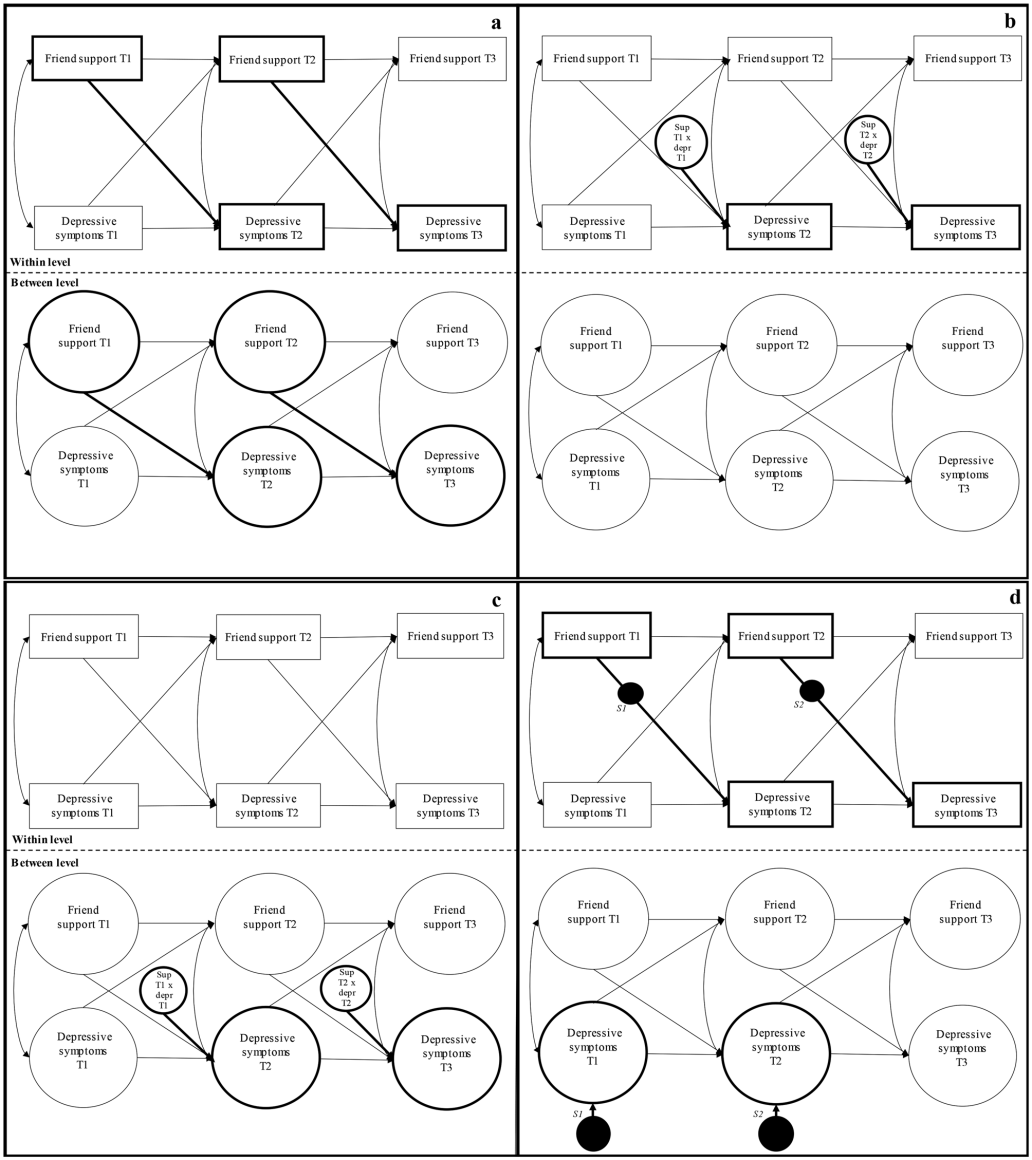
Illustration of the Hypothesized Associations between Friend Support and Depressive Symptoms on a Within- and Between-level and the Possible Moderations of Initial Levels of Depressive Symptoms on these Associations. Note. The figures are a graphical representation for clarification not an exact statistical representation. Multilevel model 1a shows the main effect model where friend support is associated with the subsequent development of depressive symptoms within- and between-dyads. Model 1b shows within-level moderation. Model 1c shows between-level moderation. Model 1d shows cross-level moderation (i.e., dyadic effect on individual level development). Squares are observed variables. Open circles are continuous latent variables (i.e., between-level support and depressive symptoms that vary across dyads). Black dots S1 and S2 represent random slopes of the effect of individual level support on subsequent depressive symptoms, that – on the between-level—vary across dyads. Single-headed arrows are path estimates and double-headed arrows are (residual error) correlations. Bold lines represent the paths of interest for the specific hypothesis. For readability, the covariate gender which was added at the between-level in all models is not depicted in the figure

The research questions are assessed in four models. The first model aims to decompose within-dyad main effects of friendship support on depressive symptoms from between-dyad main effects. To this end, an auto-regressive cross-lagged (ARCL) path model was specified to examine the association of friend support with the development of depressive symptoms over time on the within and the between level, while adjusting the estimates for reverse effects (i.e., the influence of depressive symptoms on support over time; model 1, see Fig. 1a).

Next, to test for moderation by depressive symptom levels in the link between support and subsequent depressive symptoms, we tested three interaction models (see Fig. 1b–d). The first moderation model (model 2, see Fig. 1b) considered within-level moderation in the ARCL model. In this model it is tested whether initial differences in individually-experienced depressive symptoms at year *t* would moderate the association between individual differences in perceived friendship support at year *t* and individual differences in individual adolescents’ depressive symptoms from year *t* to year *t* + 1. To this end, an interaction effect of depression x friendship support at the individual level was added to the ARCL model.

The second moderation model (model 3, see Fig. 1c) considered a between-level moderation in the ARCL model, testing whether dyadic differences in depressive symptoms at year *t* would moderate the association between support at year *t* and depressive symptoms from *t* to *t* + 1 across friendship dyads. To this end, an interaction effect between depressive symptoms and friendship support was estimated at the between level.

The third and final moderation model (model 4, see Fig. 1d) considered a cross-level moderation in the ARCL model. In this model the level of depression experienced by dyad as a whole at year *t* might affect individual differences in the association of friendship support at year *t* and subsequent depressive symptoms from *t* to *t* + 1. To this end, we specified a random slope at the between level, where individual depressive symptoms at *t* + 1 were regressed on individual support at *t*. This random slope was then regressed on dyadic depressive symptoms at year *t* on the between level. To decompose potential significant interaction effects, we probed the effect of friendship support on subsequent depressive symptoms at high (*M* + 1 *SD*) and low levels of initial depressive symptoms (*M* – 1 *SD*) (Preacher et al., 2006).

Equality constraints were imposed on all time paths for reason of parsimony, because we have no reason to assume that the magnitude of our associations would differ over the three waves measured in our study (Garcia et al., 2015). Model fit of our final model was assessed using the comparative fit index (CFI, acceptable fit ≥ 0.95), the root mean square error of approximation (RMSEA, acceptable fit ≤ 0.06), and the standardized root mean squared residual (SRMR; acceptable fit < 0.08) at the within and the between level (Hu & Bentler, 1999). The variables were standardized to avoid potential multicollinearity problems in the moderation models and to ease interpretation. Parameter estimates were controlled for possible main effects of gender (on the between-level, as we included same-gender dyads only).

## Results

### Descriptive Statistics

The means, standard deviations, range, intraclass correlations (ICCs), and Pearson correlations of depressive symptoms and best friend support at the three measurement points are presented in Table 1. The mean scores and range for depressive symptoms for the three measurement points are consecutively 14.20 (0–50), 11.75 (0–57) and 12.16 (0–54). For depressive symptoms, the ICCs were 0.04 (wave 1), 0.13 (wave 2), and 0.26 (wave 3). For friend support, ICCs were 0.39 (wave 1), 0.39 (wave 2), and 0.45 (wave 3). This shows that within dyads the scores of both friends on support and depressive symptoms are correlated, indicating a need for multilevel analysis (Peugh, 2010). Pearson correlations of depressive symptoms and perceived support scores at adjacent time points (auto-correlations) were significant and positive across time. Furthermore, friend support at all three waves was negatively correlated with the first wave of depressive symptoms (see Table 1).

**Table 1:** Means, standard deviations, range, intraclass correlation and Pearson correlations among study variables

Variable	*M*	*SD*	*Min–Max*	*ICC*	1	2	3	4	5
1. Depressive symptoms T1	14.20	10.47	0–50	.04	-				
2. Depressive symptoms T2	11.75	10.96	0–57	.13	.58**	-			
3. Depressive symptoms T3	12.16	11.14	0–54	.26	.53^**^	.70**	-		
4. Friend support T1	19.57	5.06	0–32	.39	-.15^**^	-.00	.06	-	
5. Friend support T2	19.22	5.08	5–32	.39	-.12^**^	-.06	.04	.56**	-
6. Friend support T3	18.23	5.80	0–32	.45	-.11*	.04	.02	.40**	.62**

N=226

*p < .05; **p < .01; ***p < .001

### Associations between Best Friend Support and subsequent Depressive Symptoms

**Fig. 2:**
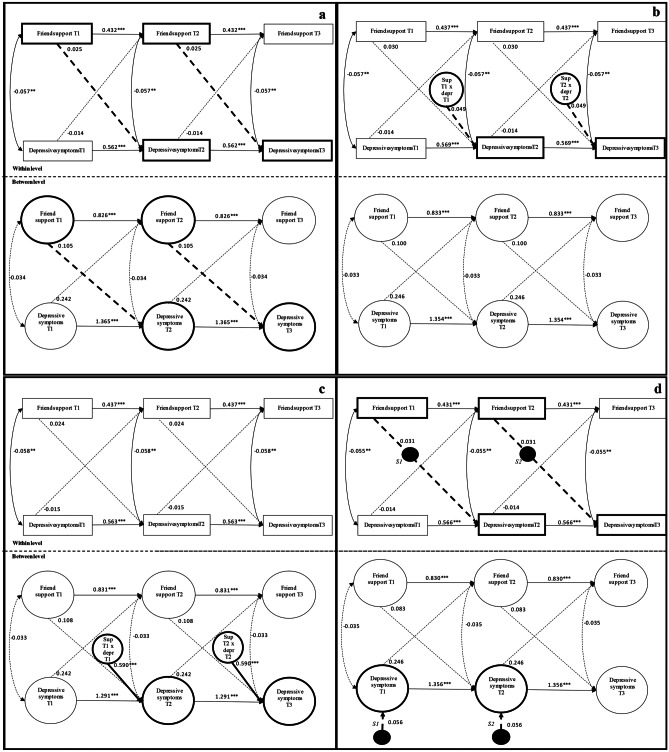
Illustration of the Associations and the corresponding estimates between Friend Support and Depressive Symptoms on a Within- and Between-level and the Possible Moderations of Initial Levels of Depressive Symptoms on these Associations. Note. The figures are a graphical representation for clarification not an exact statistical representation. Multilevel model 2a shows estimates for the main effect model. Model 2b shows estimates for the within-level moderation model. Model 2c shows estimates for the between-level moderation model. Model 2d shows estimates for the cross-level moderation model Squares are observed variables. Open circles are continuous latent variables (i.e., support and depressive symptoms on the between level that vary across dyads). Black dots S1 and S2 represent random slopes of the effect of individual level support on subsequent depressive symptoms, that – on the between-level—vary across dyads. Single-headed arrows are path estimates and double headed arrows are (residual error) correlations. Bold solid lines represent significant paths of interest for the specific hypothesis and bold dashed lines represent non-significant paths of interest for the specific hypothesis. Regression coefficients are standardized. For readability, the covariate gender which was added at the between-level in all models is not depicted in the figure *p < 0.05, **p < 0.01, ***p <0.001.N=226

We fitted the four models to test the nature of the associations between friendship support and depressive symptoms within adolescent friendship dyads. Results of all tested models are depicted in Fig. 2a–d.

In model 1 (Fig. 2a), we first tested for direct associations of best friend support and subsequent depressive symptoms at the within- and between-dyads level. Results of this model showed no significant within-dyad (*B*_within_ = 0.025, *SE* = 0.037, 95% CI = -0.049 – 0.098, *p* = 0.512) or between-dyad associations (*B*_between_ = 0.105, *SE* = 0.071, 95% CI = -0.034 – 0.244, *p* = 0.137) of support at *t* with subsequent depressive symptoms at *t* + 1.

Next, we tested the three possible moderating effects of initial levels of depression on the association of best friend support with depressive symptoms one year later. To this end, we tested moderation at the within-level (model 2; Fig. 2b), between-level (model 3; Fig. 2c) and cross-level (model 4; Fig. 2d). Results showed that the within-level interaction (model 2; Fig. 2b) and cross-level interaction (model 4; Fig. 2d) estimates were not significant (*B*_within-level interaction_ = 0.049, *SE* = 0.030, 95% CI = -0.011 – 0.108, *p* = 0.109; *B*_cross-level interaction_ = 0.056, *SE* = 0.155, 95% CI = -0.248 – 0.360, *p* = 0.718). Results of model 3 (Fig. 2c) showed a significant between-dyads moderation effect of initial level of depressive symptoms at *t* in the association of best friend support at *t* and subsequent depressive symptoms from *t* to *t* + 1 (*B* = 0.590, *SE* = 0.161, 95% CI = 0.274 – 0.906, *p* < 0.001). Model fit indices of model 3 were CFI_within_ = 0.960, CFI_between_ = 0.999, RMSEA_within_ = 0.060, RMSEA_between_ = 0.009, SRMR_within_ = 0.068, SRMR_between_ = 0.201. Overall, we deemed model fit acceptable.

**Fig. 3:**
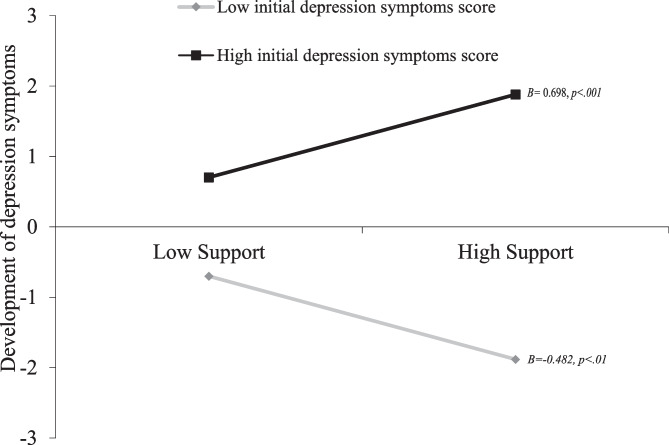
Illustration of the Between-level Model Depicting the Between-level Association of Dyadic Depressive Symptoms over Time as Moderated by the Interaction Term Friend Support x Depressive Symptoms. Note. Bs are standardized Bs

The results of probing the interaction term are depicted in Fig. 3. Results showed that when dyads experience low initial levels of depressive symptoms (1*SD* below the mean) dyads characterized by higher levels of support showed a relative decrease in depressive symptoms the next year when compared to dyads characterized by lower levels of support (*B* = -0.482, *SE* = 0.183, 95% CI = -0.841 – -0.124, *p* = 0.008). When experiencing more depressive symptoms (1*SD* above the mean) dyads characterized by higher levels of support showed a relative increase in dyadic depressive symptoms the next year compared to dyads characterized by lower levels of support (*B* = 0.698, *SE* = 0.156, 95% CI = 0.363 – 1.032; *p* < 0.001).

## Discussion

In this study, adolescents and their best friend from a relatively well-functioning Dutch sample were followed longitudinally across two years (three waves). The current study focused on their development of support and depressive symptoms, with the goal to unravel individual from dyadic level effects of the association between best friend support and subsequent depressive symptom development. Results showed no associations at the individual level. However, dyadic level associations of support with subsequent depressive symptom development were found, and this association depended upon the initial levels of depressive symptoms experienced by the dyad. That is, for dyads with few depressive symptoms, higher levels of dyadic support predicted that both members of the dyad would show relative decreases in depressive symptoms over time when compared to dyads with lower levels of support. The opposite was found for dyads characterized by more depressive symptoms. That is, for these dyads higher levels of support predicted relative increases in depressive symptoms over time for both members of the dyad, compared to dyads with lower levels of support.

Our findings on the absence of a main effect of support on adolescent depressive symptoms concur with previous studies that found no associations between friendship support and depression symptom development (Bámaca-Colbert et al., 2017; Ling et al., 2022; Lyell et al., 2020). However, several other studies did find associations between close friend support and depressive symptoms (Finan et al., 2018; Rueger et al., 2016). Because these previous studies did not distinguish individual from dyadic level effects, it is not possible to determine at which level effects actually occur. Therefore, it is possible that effects found in other studies are attributed to individual processes when in reality dyadic processes might be involved.

Furthermore, our finding that the association of support with subsequent depressive symptoms depended upon the initial levels of depressive symptoms and that this effect was only found on the dyadic level (and not on the individual level), moves beyond previous studies in two ways. First, it shows the necessity to consider dyadic level effects when studying the association between friendship support and subsequent depressive symptoms. In fact, in our study, no associations of support and subsequent depressive symptoms were found at the individual level. Although previous studies did not differentiate between dyadic and individual effects, some studies have suggested that dyadic processes such as depression contagion via peer influence or selection effects might play a role in the development of depressive symptoms within adolescent friendships (Boersma-van Dam et al., 2019; Brechwald & Prinstein, 2011; Giletta et al., 2011; Van Zalk et al., 2010). The present study confirmed this suggestion and emphasizes that part of the support-depression association may be due to either selection or within dyad processes, or both, that may make friendship dyads similar in the appreciation of support in their experienced mental state (Mankowski & Wyer, 1996; Van Zalk et al., 2010).

The second way our results moved beyond the current literature regards the nature of the dyadic level association. Although results need to be replicated first before drawing firm conclusions, our finding that the effect of best friend support on subsequent dyadic depressive symptom development was different for dyads with more depressive symptoms versus dyads with few initial dyadic depressive symptoms (i.e., initial dyadic depression levels moderate the association), is in line with previous theoretical suggestions (Gariepy et al., 2016; Pfeiffer et al., 2012). For dyads with low initial levels of depressive symptoms, dyadic support seems to have a protective effect on depressive symptom development of the dyad. This finding is supported by previous theoretical considerations and empirical evidence from two meta-analyses (Chu et al., 2010; Cohen & Wills, 1985; Rueger et al., 2016). Both meta-analyses found weak associations between higher levels of friend support and higher levels of well-being or lower levels of depressive symptoms; our study implies that these effects may be dyadic and possibly dependent on initial depression levels. Friendship support might ease emotional distress and improve mental well-being, but only when symptoms are mild.

The opposite was found for dyads with relatively more depressive symptoms. For these dyads, support was associated with a relative increase in depressive symptoms over time. Recent studies have aimed to explain this seemingly counterintuitive finding by the process of ‘co-rumination’. Co-rumination is a maladaptive communication style between friends, used to regulate their emotions. When friends co-ruminate, they discuss a problem they are dealing with thereby rehashing the problem and excessively focus on their negative feelings and emotions instead of possible solutions (Rose, 2002; Rose et al., 2007). At first, co-ruminating tends to make friends closer, as it is associated with perceived increased friendship quality and increased support (Ames-Sikora et al., 2017; Rose et al., 2007). However, co-rumination might also exacerbate adolescents’ depression experiences. Indeed, the mutual tendency to dwell on problems within friendship dyads has been associated with (increasing) depressive symptoms (Rose et al., 2007, 2014). Although never tested before (to our knowledge), one might speculate that co-rumination may manifest particularly in friendships characterized by higher depression levels. Similar to rumination, where individuals suffering from depression dwell on their problems internally (Nolen-Hoeksema, 1991), co-rumination might be characteristic of friends who show more depressive symptoms and less so of friends with relatively few depressive symptoms. As co-rumination goes hand in hand with friend support (Ames-Sikora et al., 2017; Hankin et al., 2010; Rose et al., 2007, 2014), this might explain why support from friends in certain instances might exacerbate depressive symptoms.

Thus, the current study showed—in a sample with generally mild depressive symptoms—that in order to understand processes that may explain why friendship support associates with depressive symptoms, the dyad as a whole should be considered as well as initial depression levels experienced by friends. It is important to note that all results were controlled for gender as previous literature showed mixed support for gender differences in the levels of and associations between friend support and experienced depressive symptoms (Chu et al., 2010; Gregory et al., 2020; Rose & Asher, 2017; Rose & Rudolph, 2006; Rueger et al., 2010, 2016).

Although not of main interest to our study and not officially tested, we also found that friends seem to become more alike in their level of depressive symptoms over time (as indicated by increasing ICCs). This finding is in concurrence with other studies (Boersma-van Dam et al., 2019; Schwartz-Mette & Smith, 2018; Van Zalk et al., 2010) and emphasizes that early monitoring of friends with elevated depression symptoms is warranted.

### Limitations

Two important limitations of the current study should be noted. First, the longitudinal nature of the data collection with the complex full-family design and the inclusion of only stable, same-gender friendships across the studied period may have resulted in a rather homogenous sample that may not generalize to the broader Dutch population. For instance, the sample included an over-representation of adolescents from families with a relatively high socioeconomic status and included very few adolescents from other ethnic backgrounds than Dutch. Although two recent studies that compared levels of depressive symptoms between a) native Dutch and minority youth and b) children living in the Netherlands from lower and higher socioeconomic backgrounds found no differences in depressive symptom levels for these youth in early adolescence (Buil et al., 2022; Horoz et al., 2022), some studies in other countries did find such differences (Brown et al., 2007; Wickrama et al., 2009). Moreover, it is expected that approximately 4% of adolescents in a western society have clinically elevated levels of depressive symptoms, which is 50% higher as in our sample (2.7%) (Costello et al., 2006; Keyes et al., 2019). Therefore, caution is warranted when generalizing findings.

Second, both best friend support and depressive symptoms were measured via self-reports. Although one might argue that subjectively perceived support and depressive symptoms are (most) important when studying the association between friendship support and subsequent depressive symptoms, the experienced level of depressive symptoms might influence the perception of friend support. Therefore, future studies are encouraged to replicate our findings including more objective measures of support and depressive symptoms. Lastly, we used a correlational design in our longitudinal study, and our findings by no means imply causal effects of support on depressive symptom development.

### Implications and Future Research

To understand the association between friendship support and subsequent adolescent depressive symptoms it is recommended that future research takes on a multilevel approach and searches for associations at all levels. Also, future research could be directed at understanding why support and depressive symptoms are associated on a dyadic level and why friendship dyads seem to become more alike in their depressive symptoms. It could consider processes of selection and within dyad processes that may underlie the association of friendship support and subsequent dyadic depressive symptoms over time. For instance, it might be that some dyads have a shared higher susceptibility to peer influence that causes similarity in their depressive symptoms.

Furthermore, it is recommended that researchers include gender as a variable of interest and test for potential differential effects for different genders. Although previous research is somewhat mixed on the effect of gender on the association between friend support and depressive symptoms, studies consistently show that girls are twice as likely to develop depressive symptoms than boys (Salk et al., 2017). Recent research considers co-rumination to be one of the mechanisms associated with this heightened risk (Rose, 2002; Stone et al., 2011). Co-rumination seems to be associated with more depressive symptoms regardless of gender (Rose, 2002; Spendelow et al., 2017), however several studies report girls to engage more in co-rumination than boys, resulting in higher rates of depression for girls (Felton et al., 2019; Rose, 2002; Rose et al., 2007; Spendelow et al., 2017). Thus support within girl-dyads might be characterized more by co-rumination than support within boys-dyads and therefore associated with more depressive symptoms for girls.

Moreover, studies might want to investigate the effects of support in samples of adolescents with clinical depressive disorders. Interpersonal theories of depression and empirical research suggest that adolescents with elevated depressive symptoms are less likely to form stable friendships and experience a decrease in support from friends over time (Coyne, 1976; Ren et al., 2018; Rudolph et al., 2008). Because adolescents suffering from clinical depression already experience more difficulty getting support from friends than their peers with few depression symptoms, it would be interesting to examine how the friend support they do experience is related to their depressive symptoms from a dyadic perspective. It could be that adolescents with more severe depression symptoms experience additional difficulties because in the friendships they (still) have the support is related to even higher depressive symptoms.

Lastly, future research should focus how the results generalize to different sources of social support as each of the different sources of support might have their own specific contribution to adolescent depression development. For example, previous studies found that adolescents who perceive (increasing) parental support (Gariepy et al., 2016; Stice et al., 2004), sibling support (Finan et al., 2018) or teacher support (Reddy et al., 2003) showed decreases in depressive symptoms. Also the unique contribution of each source of support should be taken into account as support is almost always given in the context of other sources of support and possible compensatory effects might affect the results (Gregory et al., 2020; Milevsky & Levitt, 2005).

If the findings are replicated in follow-up studies, the following practical implications can be considered. It may be an option for preventive and monitoring measures to incorporate dyadic features and differentiate between dyads experiencing relatively many or few depressive symptoms. Seeking support from peers is often encouraged in universal prevention programs or incorporated in interventions targeting adolescents already suffering from depressive symptoms (Ali et al., 2015; Jones et al., 2018). National and international campaigns, such as ‘hey het is ok’ – initiated by the Dutch government in 2021—or the WHO’s ‘Depression: let’s talk’ campaign—that stimulate interpersonal communication about depression, encourage people to seek support from friends, and encourage friends to listen to and assist their friend with elevated depression symptoms, might benefit from incorporating information about *how* friends can provide healthy support (i.e., advise that goes beyond ‘listening’). Without nuance, such campaigns might be counter-effective and actually might do more harm than good for some friends. This is of particular importance because adolescents themselves state to prefer informal help from friends and experience barriers when seeking professional help (Singh et al., 2019). Moreover, if follow-up studies indeed show that these processes occur systematically at a dyadic level, conventional treatment programs for depressive symptoms, such as cognitive behavioral therapy (CBT; David-Ferdon & Kaslow, 2008) and interpersonal psychotherapy (IPT; David-Ferdon & Kaslow, 2008), might also benefit from adding dyadic aspects to their therapies. For example, these programs could provide psychoeducation on how to seek and provide healthy support and on how to interact with friends in a constructive and helpful way. It would be worthwhile to consider incorporation of friends into a CBT or IPT program and provide therapy on the level of friendship dyad or provide group therapy that includes friendship dyads.

In sum, our results indicate that for adolescent friends who both experience relatively few depressive symptoms, promoting supportive dyadic relations could be beneficial. However, adolescents who form a friendship dyad characterized by relatively more depressive symptoms compared to the other adolescents in our sample, support from a best friend might exacerbate symptoms. If replicated, this potential contagion effect of the friendship might be considered in the prevention and treatment of an adolescent with depressive problems.

## Supplementary Information

Below is the link to the electronic supplementary material.

Supplementary file1 (DOCX 38 KB)

## Data Availability

The dataset of the RADAR study containing the annual data stored at DANS was used. Data were retrieved from: Research on adolescent development and relationships (young cohort)—EASY (https://www.knaw.nl).

## Code Availability

Mplus code files available at OSF via: https://osf.io/87zg9/?view_only=a2cf8bbed2784b608bc1257300e5b772
