# Supplementary material for: Perceived Support from Best Friends and Depressive Symptoms During Adolescence: Disentangling Personal from Dyadic Level Effects
Source: Res Child Adolesc Psychopathol. 2022 Dec 19;51(4):469–83. doi: 10.1007/s10802-022-00999-x (PMC10017641; doi:10.1007/s10802-022-00999-x)
Supplement: Supplementary file 1 — Supplementary file1 (DOCX 38 KB) [file 10802_2022_999_MOESM1_ESM.docx]

**Online Resource 1**

*Differences between Boys and Girls Mean level Depressive symptoms and Friend Support per Wave*

| Depressive symptoms | *n* | Mean | SD | t | df | *p* |
| --- | --- | --- | --- | --- | --- | --- |
| T1 boys | 274 | 13.38 | 10.25 | -2.07 | 450 | 0.039 |
| T1 girls | 178 | 15.46 | 10.71 |  |  |  |
| T2 boys | 274 | 9.64 | 9.47 | -4.95 | 310.56 | 0.000 |
| T2 girls | 178 | 14.99 | 12.25 |  |  |  |
| T3 boys | 274 | 9.28 | 8.96 | -6.713 | 291.24 | 0.000 |
| T3 girls | 178 | 16.60 | 12.64 |  |  |  |
| Friend Support |  |  |  |  |  |  |
| T1 boys | 274 | 18.34 | 4.94 | -6.76 | 450 | 0.000 |
| T1 girls | 178 | 21.48 | 4.66 |  |  |  |
| T2 boys | 274 | 17.83 | 4.97 | -7.67 | 450 | 0.000 |
| T2 girls | 178 | 21.37 | 4.48 |  |  |  |
| T3 boys | 274 | 16.93 | 5.55 | -6.122 | 450 | 0.00 |
| T3 girls | 178 | 20.23 | 5.65 |  |  |  |
